# Synthesis of ZnO Nanoflower Arrays on Patterned Cavity Substrate and Their Application in Methylene Blue Degradation

**DOI:** 10.3390/ma16072647

**Published:** 2023-03-27

**Authors:** Xin Zhao, Ching-Shan Wang, Ni-Ni Chou, Fang-Hsing Wang, Cheng-Fu Yang

**Affiliations:** 1 School of Information Engineering, Shanghai Zhongqiao Vocational and Technical University, Shanghai 201514, China; 2 Graduate Institute of Optoelectronic Engineering, National Chung Hsing University, Taichung 402, Taiwan; 3 Department of Chemical and Materials Engineering, National University of Kaohsiung, Kaohsiung 811, Taiwan; 4 Department of Aeronautical Engineering, Chaoyang University of Technology, Taichung 413, Taiwan

**Keywords:** lift-off, template, patterned sapphire substrate, ZnO nanoflower arrays, methylene blue degradation

## Abstract

A novel method was proposed to fabricate a ZnO seed layer with a protrusion and matrix structure, and then ZnO nanorods could be synthesized on it using the hydrothermal method to form ZnO nanoflower arrays (NFAs) easily. A patterned sapphire with a matrix cavity was used as the template, ZnO gel was deposited on the multilayer substrates using spinning coating, and the prepared seed layer with a protrusion and an array-patterned structure was moved to a Si substrate using the lift-off method. Because the ZnO seed layer exhibited a matrix and protrusion structure, ZnO nanorods were grown vertically downwards and formed ZnO NFAs. The XRD patterns resulting from analyses showed that the diffraction peaks of the five growth directions of ZnO NFAs increased as growth time increased. Furthermore, SEM and FIB analyses indicated that the length, width, aspect ratio, and total surface area of ZnO NFAs grown on the transferred seed layer increased as the synthesis time increased. Different ZnO NFAs synthesized for varying synthesis times were used to investigate methylene blue degradation, with the effect of ZnO NFAs on methylene blue degradation determined using the Beer–Lambert law. Our results demonstrate that the effect of ZnO NFAs on methylene blue degradation was enhanced with increasing synthesis time.

## 1. Introduction

Numerous process methods are proposed to synthesize ZnO nanomaterials with diverse crystal morphologies. For instance, a homogenous precipitation method utilizing NH_4_OH and urea as precipitation agents was employed to grow ZnO with needle-like microstructure and disarray structure [1]. Zhang et al. utilized different precursors, solvents, and reaction parameters to synthesize ZnO with diverse morphologies, such as prickly-sphere-, flower-, rod-, and prism-like shapes [2]. Chen et al. employed a hydrothermal method to grow ZnO nanowires (nanorods); they found that during the synthesis process, the face direction of the substrate with the seed layer, as well as the deposition time, influenced the surface morphologies of synthesized ZnO nanomaterials [3]. The synthesized ZnO nanostructures exhibited three morphologies: nanorods, beautiful chrysanthemum-like nanoflowers, and irregular plate-structured films.

In the past, template technology was an essential method for synthesizing one- and two-dimensional nanomaterials, and several materials were investigated as templates, such as polycarbonate [4], anodic aluminum oxide [5], and polystyrene [6], among others. When used as templates for growing one-dimensional and two-dimensional nanomaterials, the synthesized materials can be filled into the holes via electroplating, chemical vapor deposition, sol–gel, etc. As a result, the diameters of the nanoholes determine the diameters of the ZnO nanorods, which possess a polycrystalline structure. Wang et al. discovered that ZnO can be synthesized into nanowires or nanotubes by varying the electrophoretic voltage during the growth process [7]. In 2002, Zheng et al. proposed using zinc nitrate as the electrolyte and an alumina substrate as the working electrode [8]. In this study, a reference electrode was applied with a voltage of 1 V, and zinc foil was placed on the auxiliary electrode to deposit zinc oxide into the nanopores of aluminum oxide.

The hydrothermal synthesis method is a low-temperature technology that enables the growth of metal oxides at temperatures below 100 °C. The crystallization of metal oxides is achieved by controlling various synthesis parameters of the aqueous solution, such as temperature, concentration, and pH value. In the case of synthesizing zinc oxide, salts such as zinc nitrate hexahydrate (Zn(NO_3_)_2_·6H_2_O) provide the source of Zn ions, while hexamethylenetetramine (HTMA) hydrolyzes to produce ammonia (NH_3_) and formaldehyde (HCHO) [1,2,3]. The main role of NH3 is to create an alkaline environment that allows Zn^2+^ and OH^-^ ions to combine and form Zn(OH)_2_, and it serves as the precursor for growing ZnO crystals. The resulting product is deposited on the seed layer and undergoes thermal dehydration to form a solid-phase product, which can then grow along the direction of the ZnO crystal structure.

The growth of ZnO-based nanostructure materials involves two main chemical reactions: hydrolysis and dehydration. These reactions are responsible for the hydrolysis reaction procedure and the dehydration reaction procedure, respectively [9,10]. From the equilibrium reaction formulas in these references, we can observe that the direction of the reaction is controlled by the synthesis temperature, reaction time, and concentrations of reactants. Typically, the concentration of reactants determines the density of one-dimensional ZnO-based nanostructures. On the other hand, the synthesis time and temperature can regulate the surface morphology, including the width and length, of synthesized ZnO nanorods. The first primary objective of this study is to investigate the impact of synthesis time on the growth of ZnO nanostructures using the hydrothermal method and an investigated novel seed layer. Additionally, we show that this investigated method could easily synthesize ZnO nanorods in the form of ZnO nanoflower arrays (abbreviated as NFAs). A significant finding of this research is the development of an inexpensive technique to create a ZnO seed layer with a matrix and protrusion structure for synthesizing ZnO NFAs using a combination of template technology and the hydrothermal method for synthesizing one-dimensional ZnO-based nanomaterials. This approach involves the uses of patterned sapphire as templates and lift-off as the technology to create ZnO NFAs [11,12]. The synthesis results of ZnO NFAs were analyzed by varying the synthesis time while keeping the other parameters constant [13].

Because of the rapid progression of industrialization, environmental pollutions have become an increasingly serious issue, drawing considerable attention. Methylene blue, a dye and medication, is released from textile factories as effluent dye compounds, and is an important source of eutrophication and pollution in aquatic environments and ecosystems. Many materials have been investigated for their ability to reduce the concentration of methylene blue in water [14,15,16], and ZnO-based materials have also been found to have photocatalytic properties that can degrade methylene blue in water [17]. Our second important objective is that we used a simple method to examine the morphologies and calculate the growth-related characteristic parameters of ZnO NFAs synthesized at different times. The third important object is that we also used synthesized ZnO NFAs to process the methylene blue degradation and found that ZnO NFAs grown for longer periods of time exhibited enhanced photocatalytic effects.

## 2. Materials and Methods

**Figure 1:**
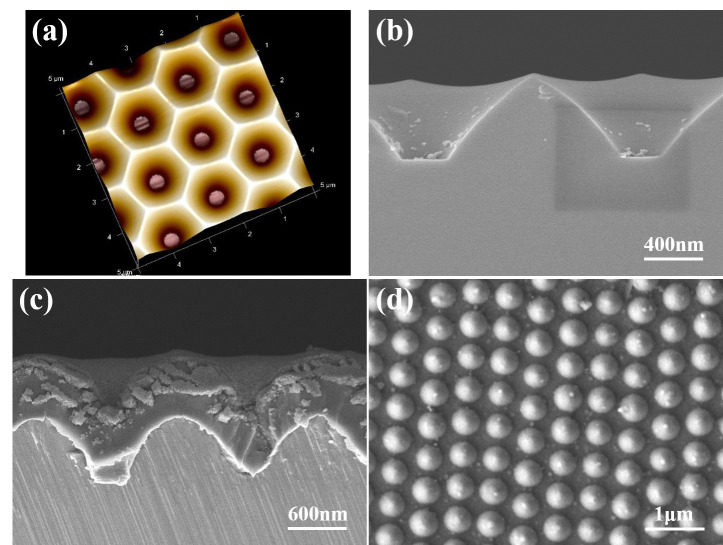
(**a**) A schematic diagram and (**b**) a cross-sectional image of a two-inch sapphire substrate. (**c**) A cross-sectional image of the deposited seed layer, and (**d**) a top view of a prepared patterned seed layer.

Zn(CH_3_COO)_2_-2H_2_O, C_2_H_7_NO, and CH_3_OCH_2_CH_2_OH were used to prepare a 0.2 M solution of Zn^2+^ ions, which was stirred at 60 °C for 2 h. The solution was left standing for 48 h to obtain a ZnO gel solution. A 2-inch sapphire substrate with a patterned cavity, as shown in Figure 1a,b, was used as template and the Al sacrificial layer was deposited on it. The patterned cavity had a bottom width of 0.37 μm and an average length of 0.48 μm. A 120 nm thick layer of Al was deposited using the evaporation method, and the sapphire template with the Al sacrificial layer was annealed at 500 °C for 1 h with a pressure of 1.2 bar using 90% Ar + 10% H_2_. ZnO gel was then spin-coated onto the surface of the sapphire template, using a rotation speed of 2000 rpm and a coating time of 30 s. Then, the ZnO gel was coated on Al-coated template and baked for 10 min at 300 °C to dry and harden the coating of ZnO gel. In order to be able to obtain sufficient thickness of ZnO seed layer, we repeated the coating and baking processes six times. A focused ion beam (FIB) system was used to observe the deposited seed layer, as shown in Figure 1c; only nanoscale particles were formed. An optical OE-6370HF AB glue was used as a carrier and coated on the ZnO thin layer, and the prepared multilayer materials were removed by using the lift-off method and imprint lithography. The Al sacrificial layer was etched using a solution of K_3_Fe(CN)_6_:KOH:H_2_O (10 g:1 g:100 mL). The ZnO seed layer was adhered onto a p-type Si substrate, which used OE-6370HF AB glue as adhesive. Then, a seed layer with a patterned and protrusion array structure was created, as depicted in Figure 1d. ZnO NFAs were synthesized using a 0.2 M solution of Zn(CH_3_COO)_2_-2H_2_O at 90 °C for different synthesis times (10, 20, 30, and 60 min). The surface morphologies of prepared seed layer and synthesized ZnO NFAs were observed by a FESEM, while their crystalline phases were analyzed using an X-ray diffractometer. Cross-sectional morphologies were observed using a FIB system. The optical properties of synthesized ZnO NFAs were measured at room temperature using a Horiba Jobin iHR550 fluorescence spectrophotometer in the wavelength range of 350−650 nm, whose excitation light source was a He Cd laser at a single wavelength of 325 nm

The ZnO NFAs synthesized at different times were used in experiments to test their efficacy in degrading methylene blue. For this purpose, a methylene blue solution with a concentration of 0.5 ppm was prepared, and 20 × 20 mm^2^ ZnO NFAs were immersed in 5 mL of the solution. The ZnO NFAs and solution were then exposed to an ultraviolet (UV) light with a wavelength of 265 nm for varying durations ranging from 0 to 10 h. The degradation effect of methylene blue in the liquid was analyzed using the Beer–Lambert law, and the average value of five samples was determined to measure the variations in concentrations of the methylene blue solutions. A Hitachi U-3300 UV–Vis spectrophotometer was used to measure the optical transmittance spectra of the methylene blue liquids in the wavelength range of 200–700 nm.

## 3. Results and Discussion

**Figure 2:**
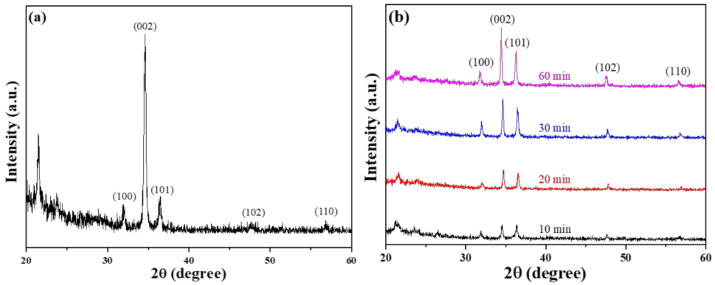
XRD patterns of (**a**) prepared ZnO seed layer and (**b**) different-time-synthesized ZnO NFAs.

The diffraction peaks of the ZnO seed layer for the (100), (002), (101), (102), and (110) planes were observed at corresponding to 2θ values of 31.9°, 34.6°, 36.5°, 47.6°, and 56.8°, as Figure 2a shows. The highest diffraction peak was observed at 34.6°, which corresponds to the (002) plane and is higher in intensity than the other four diffraction peaks. This result suggests that the characteristic of c-axis preferred orientation exists in the ZnO seed layer even at the nanoscale level. The XRD patterns of the ZnO NFAs synthesized were analyzed as a function of synthesis time, and the results are shown in Figure 2b. All the diffraction peaks presented in the seed layer ((100), (002), (101), (102), and (110) planes) were observed in all synthesized ZnO NFAs, and the diffraction intensity of the (002) plane increased significantly with synthesis time. 

XRD analysis of ZnO NFAs prepared at different synthesis times showed an increase in peak intensities corresponding to the five growth directions of zinc oxide, indicating the HCP array and wurtzite structure of the nanoflowers. Furthermore, the intensity of these peaks increased with synthesis time, indicating that growth time of ZnO NFAs is an important factor in enhancing their crystal property. Notably, a c-axis preferred orientation is not presented in ZnO NFAs, because their main growth directions are perpendicular to the matrix and protrusion ZnO seed layer, but they are not necessarily perpendicular to Si substrate and grow vertically. Instead, the grown ZnO nanorods spread out in a flower-like manner to form ZnO nanoflowers.

**Figure 3:**
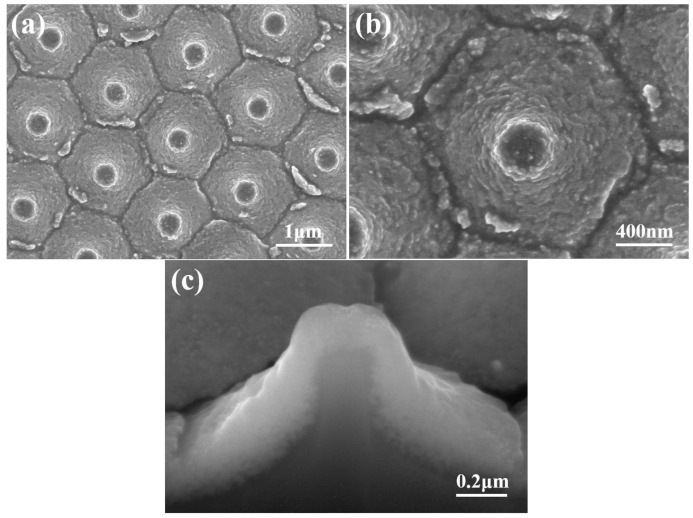
SEM images of an investigated template with (**a**) a low magnification and (**b**) a high magnification; and (**c**) the FIB cross-sectional view of the template.

SEM images of the novel template at various magnifications are presented in Figure 3a,b, while a FIB cross-sectional view is displayed in Figure 3c. These images reveal that only nanoscale particles are visible in the top view and FIB cross-sectional image. Figure 3b,c indicate that the diameter and thickness of the deposited ZnO seed layer are approximately 2 μm and 206 nm, respectively. These images also confirm that the lift-off method can successfully transfer a protrusion seed layer from a sapphire substrate on a Si substrate. Figure 3b was used to measure the average particle diameter and size of the prepared seed layer, and they were found to be approximately 365 and 32 nm. The thickness of the ZnO seed layer with a measured value of 206 nm was determined from an image obtained from a FIB, as shown in Figure 3c.

**Figure 4:**
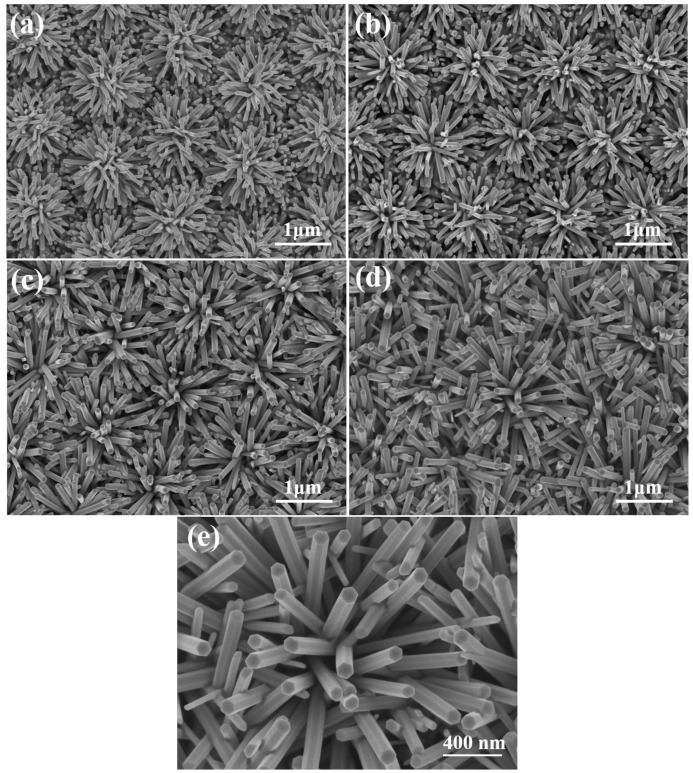
ZnO NFAs synthesized at (**a**) 10, (**b**) 20, (**c**) 30, and (**d**) 60 min. (**e**) 60 min-synthesized ZnO NFAs with higher magnification.

SEM images of ZnO NFAs with different synthesis times are shown in Figure 4. Due to the protrusion structure of the ZnO seed layer depicted in Figure 1d and Figure 3, ZnO nanorods can grow radially and perpendicularly to the seed layer, resulting in the formation of ZnO nanoflowers. As the seed layer is patterned in an array structure, the resulting ZnO nanoflowers also exhibit an array pattern. When the synthesis time was 10 min (Figure 4a), ZnO nanorods with a well-formed structure were observed, resulting in the formation of ZnO NFAs. However, for synthesis times less than 30 min, synthesized ZnO nanorods with short lengths were observed. Figure 4 demonstrates that as the synthesis time increases, both the diameters and lengths of ZnO nanorods also increase, while the density (number) of ZnO nanorods in NFAs is significantly reduced. However, as depicted in Figure 4a,b, the diameter and length of the nanorods were too thin and short, which we believe resulted in weaker diffraction intensities of the (100), (002), (101), and (102) planes. As the synthesis time increased from 10 to 20 min, Figure 4a,b illustrate a significant increase in both the diameter and length of ZnO NFAs. As the XRD patterns shown in Figure 2b are compared, the diffraction intensities of the (100), (002), (101), and (102) planes noticeably increase with longer synthesis times.

These findings indicate that the crystallization of ZnO nanorods is improved with longer synthesis times. While there was only a slight increase in both length and diameter of ZnO nanorods when we increased the synthesis time from 30 to 60 min, the pushing effect of ZnO nanorods was observed during the synthesis process, leading to more ZnO nanorods growing in an upward vertical direction to form ZnO NFAs; Figure 4c,d display these results. The results presented in Figure 4 demonstrate that the prepared ZnO seed layer facilitates the growth of ZnO nanorods in a direction perpendicular to the seed layer, resulting in the formation of ZnO nanoflowers. Furthermore, as shown in Figure 4, the diameter (D), length (L), and aspect ratio (L/D) of ZnO nanorods are relatively linked to synthesis time, when all other conditions are kept constant. The XRD patterns presented in Figure 2 suggest that even the (002) plane is the primary growth direction; the other four diffraction directions also increase with synthesis time. As the main growth directions of ZnO nanorods are perpendicular to the seed layer and spreads out in a flower-like pattern to form ZnO nanoflowers, as observed in Figure 4. The ZnO seed layer is prepared in a matrix pattern, resulting in a corresponding matrix distribution of ZnO NFAs. Therefore, ZnO nanorods are synthesized in accordance with the protrusion pattern of the seed layer and can form ZnO NFAs. Figure 4e displays the 60 min-synthesized ZnO NFAs with higher magnification and it demonstrates that all these synthesized ZnO nanorods had the structure of hexagonal prisms, and their diameters had little differences.

The uniformity of both the diameter and length of ZnO nanorods can be seen in Figure 4. The values of radius and length presented in Table 1 were employed to determine the surface area, volume, and aspect ratio of ZnO nanorods, the corresponding results are also provided in Table 1. It is worth noting that the structure of the ZnO nanorods is a hexagonal cylinder, rather than a column. When a hexagonal cylinder has a radius *r* = *a*/2 and side length *a*, its volume is ($\sqrt{75}$/8)π*a*^3^; if the radius is decreased to *r* = *a*/4 (divided into 4 cylinders) or to *r* = *a*/6 (9 cylinders), and the length of each cylinder is still *a*. As the radii are *a*/2, *a*/4, and *a*/6, the total surface areas (S) are ($\sqrt{3}$/16 + 3)π*a*^2^, ($\sqrt{3}$/16 + 6)π*a*^2^, and ($\sqrt{3}$/16 + 9)π*a*^2^, respectively, and all the total volumes are ($\sqrt{75}$/8)π*a*^3^, as Table 1 shows. All S/V ratios have been calculated and presented in Table 1, indicating that the S/V ratio increases as the diameter decreases. As the total volume (V) of ZnO nanorods in a unit area remains constant, the decrease in the diameter of ZnO nanorods leads to an increase in the density (quantity per unit area) of cylinders, as well as an increase in both the S value and S/V ratio. When ZnO nanorods have the same total volumes, their response characteristics are influenced by both their S value and S/V ratio, making them ideal for use as optical sensors or devices with an enhanced effect.

**Figure 5:**
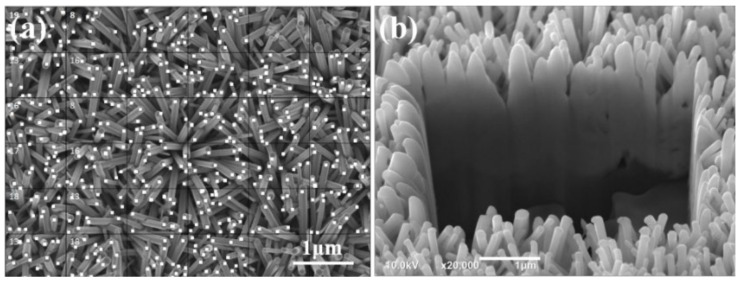
(**a**) Surface morphology used to estimate the density and (**b**) cross-sectional image used to estimate the length of ZnO NFAs.

Figure 4 illustrates the close relationship between the synthesis time and the length (L), diameter (D), and aspect ratio (L/D) of ZnO NFAs. To determine the density of ZnO NFAs in a unit area, we divided them into squares of 1 μm^2^, as Figure 5a shows. Six random squares were selected to compute the density and the average dimensions of ZnO nanorods in each area. Table 1 presents a comparison of the measured average D, H, and L/D values of ZnO NFAs synthesized at different times. Figure 4 indicates that for synthesis times of 10 and 20 min, ZnO NFAs do not completely cover the ZnO seed layer; therefore, the total surface area (S), total volume (V), and S/V ratio could not be determined for these times, and their relative parameters were excluded from Table 1. The densities of ZnO nanorods for the synthesis times of 30 and 60 min are 26 μm^–2^ and 28 μm^–2^. Figure 5b displays the cross-sectional image, which can be utilized to estimate the L value of ZnO NFAs. The L values of ZnO nanoflowers were estimated by subtracting 206 nm, which was the thickness of the ZnO seed layer. As the synthesis times were 10, 20, 30, and 60 min, the L values of ZnO NFAs were found to be 483, 718, 987, and 1500 nm, respectively, with corresponding average diameters of 38, 54, 71, and 90 nm. The calculated L/D values increased from 12.7, 13.3, 13.9, to 16.7 with an increase in synthesis time from 10, 20, 30, and 60 min. 

When the synthesis time is less than 30 min, incomplete growth of ZnO nanorods is observed, as illustrated in Figure 4a,b, and the D and L values of ZnO nanorods increased with synthesis time. For synthesized ZnO NFAs, their V values increased from 1.02 × 10^8^ to 1.02 × 10^8^ nm^3^, while S values increased from 4.56 × 10^6^ to 1.20 × 10^7^ nm^2^ with an increase in synthesis time from 30 to 60 min. As the synthesis time of ZnO NFAs increased from 30 to 60 min, their S/V ratio showed a slight increase from 4.47 × 10^−2^ to 4.49 × 10^−2^. These results demonstrate that adjusting the synthesis time can control the V and S values and S/V ratio of synthesized ZnO NFAs. We strongly believe that the synthesis time significantly affects the V and S values of synthesized ZnO NFAs, and consequently affects their PL properties. As presented in Table 2, an increase in synthesis time from 30 to 60 min led to a notable increase in all morphological parameters of synthesized ZnO NFAs, such as the average D value, L value, L/D ratio, density, S value, V value, and S/V ratio.

**Figure 6:**
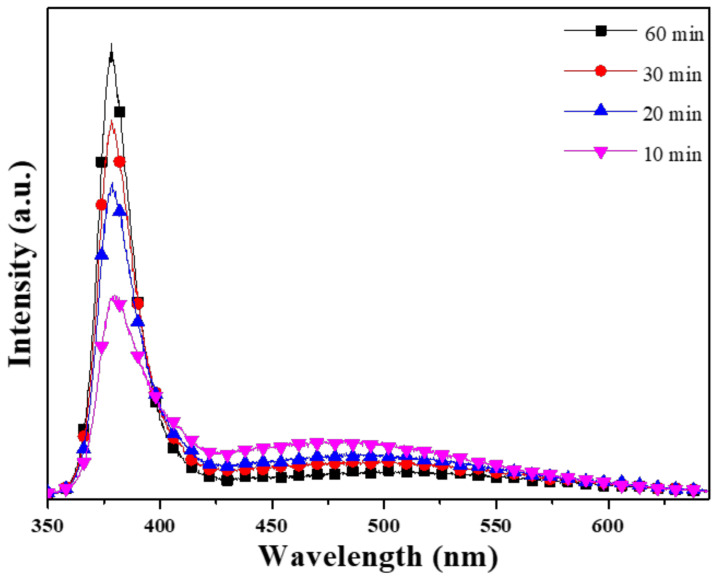
Effect of synthesis time on the PL spectra of ZnO NFAs.

The photoluminescence (PL) spectra of ZnO NFAs are shown in Figure 6, which were excited using a UV light of 325 nm. The spectrum exhibited two emission peaks: an ultraviolet (UV) emission peak centered at approximately 380 nm (I_UV_), which is a typical near-band-edge emission, which is caused by the recombination process of free excitons [18], and a green light emission (I_G_) ranging from approximately 450 to 550 nm, which is caused by the combinations of different defects existing in synthesized ZnO NFAs. Table 3 presents a comparison of the emission intensities of the two main peaks under different synthesis times. The decrease in I_G_ value indicates that the defects of ZnO NFAs decrease with increasing synthesis time, and the possible reasons for this trend are further discussed. There exist various luminescence mechanisms in ZnO-based materials. In previous studies, Lin et al. identified various defects in ZnO-based materials, including zinc vacancies (V_Zn_), interstitial zinc (Zn_i_), antisite defect (O_Zn_), interstitial oxygen (O_i_), and oxygen vacancies (V_O_), which contribute to the generation of ultraviolet (UV) light emission at around 390 nm or 3.18 eV, violet light emission at around 405 nm or 3.06 eV, violet light emission at around 414 nm or 2.99 eV, green light emission at around 521 nm or 2.38 eV, and 544 nm 2.28 eV, and near-infrared emission at around 765 nm or 1.62 eV [19]. 

Figure 6 illustrates the trend in the ultraviolet light intensity (I_UV_) increasing with growth time, while the intensity of green light (I_G_) decreases, and the sharp peaks are located at 378.8, 378.6, 378.3, and 378.4 nm as 10, 20, 30, and 60 min were used as the synthesis times. Table 3 reveals that the I_G_/I_UV_ ratio decreased as the synthesis time increased from 10 to 60 min. Furthermore, the FWHM (full width at half maximum) values for the I_UV_ peaks (located at 378.3–378.4 nm) of the PL spectra were 26.8 (371.3–398.1 nm), 20.4 (371.4–391.8 nm), 18.9 (371.6–390.5 nm), and 17.6 nm (371.4–389 nm) as the synthesis time was 10, 20, 30, and 60 min. These results demonstrate that as the synthesis time increases, the FWHM value decreases, and the emission peak becomes stronger. 

**Figure 7:**
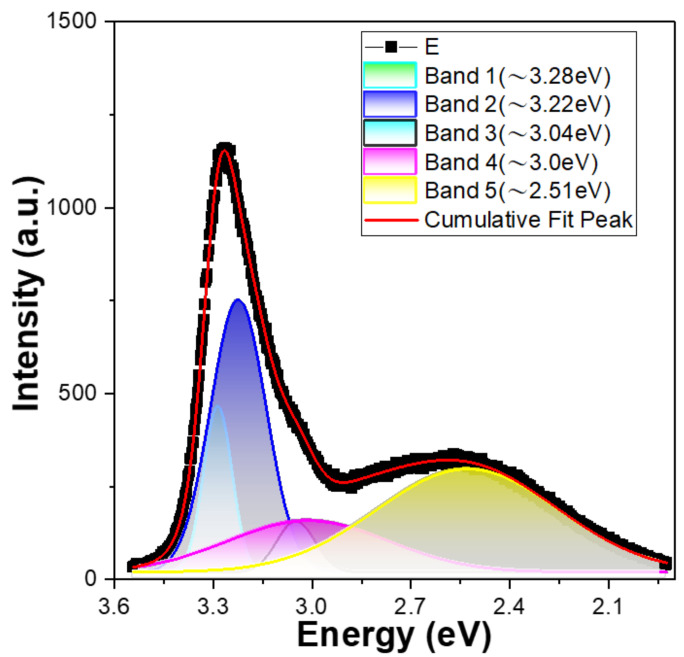
PL spectrum of 10 min-synthesized ZnO NFAs and the fitting results using the sum of five Gaussian functions.

The PL spectrum of 10 min-synthesized ZnO NFAs is shown in Figure 7, with energy plotted on the x-axis (not wavelength). When estimating the PL spectrum of 10 min-synthesized ZnO NFAs, the merged emission profile in the range of approximately 3.54–1.92 eV (350–645 nm) was fitted to a sum of five Gaussian functions located at approximately 3.28, 3.22, 3.04, 3.00, and 2.51 eV, respectively. Notably, the bandwidth and intensity of the band at 3.04 eV were small in the fitting result. Previously, Purbayanto et al. discovered that emission peaks at 3.251 and 3.327 eV are associated with donor–acceptor pair transitions and two-electron satellite transitions [20]. Their findings indicate that the 3.28 eV peak in Figure 7 is likely linked to either donor–acceptor pair or two-electron satellite transitions. The 3.22 eV peak is caused by the near-band-edge emission, while the 3.04 and 3.00 eV peaks are believed to be caused by zinc vacancies and interstitial zinc. Purbayanto et al. conducted H_2_ annealing and observed that the strong emission at 2.5 eV persisted at room temperature. They suggested that this emission was due to localized states, with oxygen vacancies (V_O_) identified as the primary localized states. Additionally, they noted that the high defect concentration was responsible for the strong emission at 2.5 eV. However, the relative energy resulting from oxygen vacancies differs from the findings of Lin et al. Consequently, the 2.51 eV peak is attributed to oxygen vacancies produced during the synthesis process. In Figure 6, it can be observed that the intensity of the green emission peak decreases as the synthesis time of ZnO NFAs increases. This indicates that the amount of oxygen vacancies in synthesized ZnO NFAs decreases as the synthesis time lengthens.

**Figure 8:**
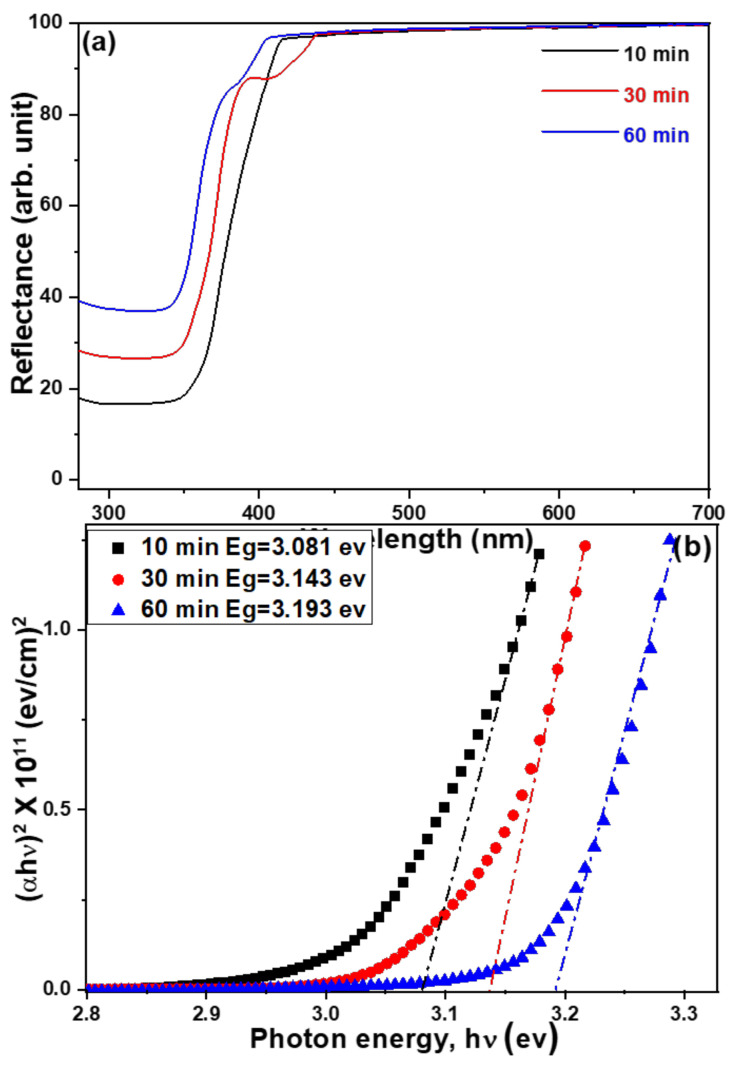
(**a**) UV–visible diffusion reflectance spectra and (**b**) Tauc plots for UV–visible reflectance spectra of ZnO NFAs using different synthesis times.

The drawings of the UV–visible diffusion reflectance spectra of ZnO NFAs for samples with different synthesis times are shown in Figure 8a and their corresponding Tauc plots are shown in Figure 8b. UV–visible diffusion reflectance spectra of ZnO NFAs reveal the optical reflectance in the UV region which can be recognized as the migration of electrons from the valance band to the conduction band [12]. These UV–visible spectra reveal that their reflectance rates increase with the synthesis time of ZnO NFAs. The calculated band gaps for samples grown with the synthesis times of 10, 30, and 60 min were 3.081, 3.143, and 3.193 eV, respectively. 

**Figure 9:**
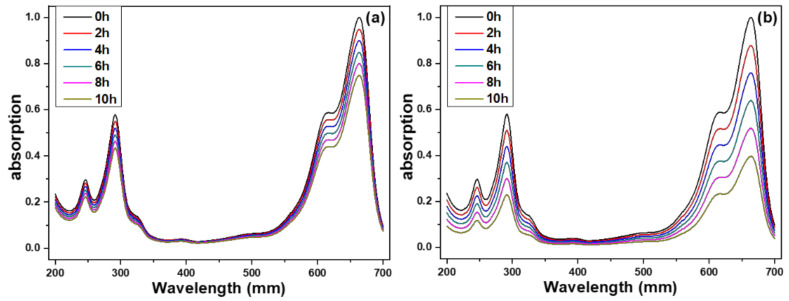
Ultraviolet–visible spectra of treated methylene blue solution under different irradiation time of UV light and different synthesis times of ZnO NFAs, (**a**) 20 min and (**b**) 60 min.

The photocatalytic activity of prepared ZnO NFAs was evaluated by an experiment examining methylene blue degradation by using UV light irradiation. The transmittance spectra of methylene blue solutions using ZnO NFAs synthesized at different times as photocatalytic materials are presented in Figure 9. Methylene blue exhibits three strong absorption bands at 246, 293, and 664 nm, with the strongest absorption occurring at 644 nm. Therefore, the intensity of the 644 nm absorption band was used as a reference for evaluating the degradation results. As shown in Figure 9, the intensities of the entire absorption spectrum and the three absorption bands of methylene blue solution gradually decreased as the UV light irradiation time increased from 0 to 10 h. For instance, when 20 min-synthesized ZnO NFAs were used as photocatalytic materials, the average intensity of the 664 nm band decreased from 94.9% to 90.0%, 84.8%, 80.1%, and 74.9% as the irradiation time of UV light increased from 2 to 10 h (Figure 9a). When using 60 min-synthesized ZnO NFAs as photocatalytic materials, the intensity of the 664 nm band decreased by 87.8% to 75.9%, 63.8%, 51.8%, and 39.6% as the UV light irradiation time increased from 2 to 4, 6, 8, and 10 h (Figure 9b). The experimental result showed that, even when the concentration of methylene blue is 5 ppm, ZnO NFAs synthesized for 30 and 60 min can effectively degrade more than 50% of the methylene blue after 10 h of exposure. These transmittance spectra of methylene blue solutions indicate the degradation effect of ZnO NFAs on the methylene blue solution. The results in Figure 9 demonstrate that different synthesis times result in ZnO NFAs having different morphologies, which in turn produce different degradation effects on methylene blue solution. 

The linear relationship between the concentration of an absorbing species and absorbance can be described using the Beer–Lambert law. It is commonly applied to understand the variations of attenuations in physical optics for rarefied gases, neutrons, and photons; it is also widely used in analytical measurements in chemistry. The law can be expressed as:

*A* = ln (*Io*/*I*) = *abc* (1)

**Figure 10:**
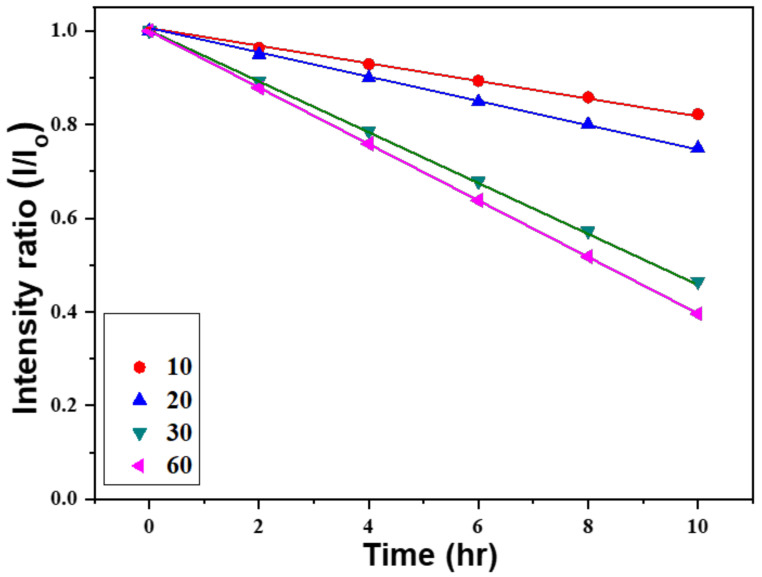
Variations of absorption intensities for methylene blue solutions under different irradiation time of UV light and synthesis time of ZnO NFAs.

In this equation, *A*, *Io*, and *I* are the absorbance, the transmitted light, and the incident light, respectively. The variables *a*, *b*, and *c* represent the absorptivity (with unit of L mol^−1^ cm^−1^), width of the quartz cell containing the detection solution (path length), and the concentration of the detected material in solution (expressed in mol L^−1^). All the measured absorption intensities at 664 nm are divided by the unmeasured intensity to obtain the normalized values. Figure 10 shows the relationship between the irradiation time of methylene blue degradation and the intensity of the absorbance peak at 664 nm. When ZnO NFAs synthesized at different times were used as photocatalysts, the changes of all the measured results can be represented by a straight-fitting line. Compared to ZnO NFAs synthesized for 10 and 20 min, 30 min and 60 min-synthesized ZnO NFAs have the larger slopes. These results demonstrate that ZnO NFAs synthesized for 30 and 60 min exhibit enhanced photocatalytic responses with respect to methylene blue degradation under UV light irradiation. As Figure 10 shows, the absorbance of the spectral peak at 664 nm decreased monotonically with the irradiation time. When using 60 min-synthesized ZnO NFAs as the photocatalyst, the fitted line obtained by the normalized measured intensities had a larger slope than when the lines were fitted by using ZnO NFAs synthesized for shorter times. This indicates that the 60 min-synthesized ZnO NFAs exhibit the optimum photocatalytic effect in methylene blue degradation. The observed variations in the morphology of ZnO NFAs are consistent with the results shown in Figure 4, indicating that an increase in the total surface area of ZnO NFAs leads to a greater degradation effect on methylene blue.

The Beer–Lambert law indicates that the residual ratio of methylene blue is directly proportional to its concentration. However, regardless of the growth time, the results in Figure 10 show that the degradation process leads to a linear decrease in the concentration of methylene blue for all ZnO NFAs. Therefore, these linearly decreasing curves can be expressed as Equation (2):

*y* = *a* + *b x* (2)

As the synthesis times of ZnO NFAs were 10, 20, 30, and 60 min, the values for *a* were 0.99957, 0.99942, 0.99956, and 0.99978, and the values for *b* were −0.01777, −0.02500, −0.05347, and −0.06033, respectively. Obviously, all the methylene blue degradation showed a linear decrease, and the longer the growth time of ZnO NFAs, the faster the rate of decrease in the concentration of methylene blue. One important evaluation parameter is the coefficient of determination, denoted R-R (R^2^) value and pronounced as “R squared”. As the synthesized times of ZnO NFAs were 10, 20, 30, and 60 min, the R-R values were 0.9995, 0.9994, 0.9999, and 0.9999, respectively. The high R-R values reveal that the linear equation matches the experimental data well.

## 4. Conclusions

In this study, the proposed method was successfully to synthesize ZnO nanorods into ZnO nanoflower arrays (NFAs). A series of experiments were successfully conducted to calculate the morphology, crystal characteristics, diameter (D), length (L), and aspect ratio of ZnO NFAs at different synthesis times. The diffraction patterns of the (100), (002), (101), (102), and (110) planes were observed in all synthesized ZnO NFAs. The intensity of diffraction pattern for the (002) plane increased significantly with increasing synthesis time. As the synthesis times of ZnO NFAs were 10, 20, 30, and 60 min, the L values were measured to be 483, 718, 987, and 1500 nm, and the corresponding average diameters were 38, 54, 71, and 90 nm, and the L/D values increased from 12.7, 13.3, 13.9, to 16.7.

The photoluminescence spectrum of ZnO NFAs synthesized for 10 min was also successfully fitted to a sum of five Gaussian functions located at approximately 3.28, 3.22, 3.04, 3.00, and 2.51 eV. These emission peaks were found to be attributed by the donor–acceptor pair or two-electron satellite transitions, the near-band-edge emission, zinc vacancies, interstitial zinc, and oxygen vacancies, respectively. These results can be used to analyze the defects residual in synthesized ZnO NFAs. As the synthesis time of ZnO NFAs increased, the photocatalytic degradation effect also increased. Specifically, when using ZnO NFAs synthesized for 60 min as photocatalytic materials and the irradiation time of UV light increased from 2 to 10 h, the intensity of the 664 nm band decreased by 87.8%, 75.9%, 63.8%, 51.8%, and 39.6%. These results indicate that the morphologies of synthesized ZnO nanorods are an important factor to affect their applications in photocatalytic degradation.

## Figures and Tables

**Table 1:** Parameters used to calculate S/V ratio of ZnO nanorods in NFAs.

Radius	r=a/2	r=a/4	r=a/6
length	h=a	h=a	h=a
surface area (S)	($\sqrt{3}$/16 + 3)πa^2^	($\sqrt{3}$/16 + 6)πa^2^	($\sqrt{3}$/16 + 9)πa^2^
volume (V)	($\sqrt{75}$/8)πa^3^	($\sqrt{75}$/8)πa^3^	($\sqrt{75}$/8)πa^3^
S/V	($\sqrt{3}$/16 + 3)/($\sqrt{75}$/8)a	($\sqrt{3}$/16 + 6)/($\sqrt{75}$/8)a	($\sqrt{3}$/16 + 9)/($\sqrt{75}$/8)a

**Table 2:** Relative results of ZnO NFAs as a function of synthesis time.

time (min)	30	60
average diameter (D, nm)	71	90
average height (H, nm)	987	1500
aspect ratio (H/D)	13.9	16.67
density (μm^−2^)	26	28
total surface area (S, nm^2^)	4.56 × 10^6^	1.20 × 10^7^
total volume (V, nm^3^)	1.02 × 10^8^	2.67 × 10^8^
S/V ratio	4.47 × 10^−2^	4.49 × 10^−2^

**Table 3:** Defect comparisons of ZnO NFAs under different synthesis times.

Time (min)	I_UV_	I_G_	I_G_I_UV_
10	1156.32	336.95	0.291
20	1803.93	263.22	0.145
30	2160.91	257.37	0.119
60	2596.79	163.10	0.062

## Data Availability

Not applicable.

## References

[1] Karthik T.V.K., Lugo V.R., Hernandez A.G., Navarro Jiménez J.L., Sanchez-Campos D., Pozos H.G., Mendoza Anaya D., Cerecedo-Sáenz E. (2019). Low temperature facile synthesis of ZnO nuts and needle like microstructures. Mater. Lett..

[2] Zhang J., Sun L.D., Yin J.L., Su H.L., Liao C.S., Yan C.H. (2002). Control of ZnO Morphology via a Simple Solution Route. Chem. Mater..

[3] Chen Y.C., Cheng H.Y., Yang C.F., Hsieh Y.T. (2014). nvestigate the Optimal Parameters in Hydrothermal Method for the Synthesis of ZnO Nanorods. J. Nanomater..

[4] Jaleh B., Hamzehi S., Sepahvand R., Azizian S., Eslamipanah M., Golbedaghi R., Meidanchi A., Fausto R. (2022). Preparation of Polycarbonate-ZnO Nanocomposite Films: Surface Investigation after UV Irradiation. Molecules.

[5] Zhang H., Li X., Hu G., Li Y. (2010). Fabrication of ZnO nanostructure within the AAO template by electrochemical deposition. J. Mater. Sci. Mater. Electron..

[6] Wang Y.C., Leu I.C., Hon M.H. (2002). Preparation of Nanosized ZnO Arrays by Electrophoretic Deposition. Electrochem. Solid-State Lett..

[7] Zhang L.D., Li G.H., Shen W.Z. (2002). Fabrication and optical properties of large-scale uniform zinc oxide nanowire arrays by one-step electrochemical deposition technique. Chem. Phys. Lett..

[8] Ahsanulhaq Q., Umar A., Hahn Y.B. (2007). Growth of aligned ZnO nanorods and nanopencils on ZnO/Si in aqueous solution: Growth mechanism and structural and optical properties. Nanotechnology.

[9] Sun Y., Riley D.J., Ashfold M.N.R. (2006). Mechanism of ZnO Nanotube Growth by Hydrothermal Methods on ZnO Film-Coated Si Substrates. J. Phys. Chem. B.

[10] Ayeleru O.O., Dlova S., Ntuli F., Kupolati W.K., Olubambi P.A. (2019). Development and Size Distribution of Polystyrene/ZnO nanofillers. Procedia Manuf..

[11] Lee C.Y., Wang C.S., Wang F.H., Liu H.W., Yang C.F. (2022). Investigations of a statistical and analysis method to find the relationship between the morphological and optical properties of ZnO nanoflower arrays. ACS Omega.

[12] Tseng H.S., Wang C.S., Wang F.H., Liu H.W., Yang C.F. (2022). A novel synthesis of ZnO nanoflower arrays using lift-off technique with different thicknesses of Al sacrificial layers on a patterned sapphire substrate. Nanomaterials.

[13] Yang C.F., Wang C.S., Wang F.H., Liu H.W., Micova J. (2022). Effect of Synthesis Time on Synthesis and Photoluminescence Properties of ZnO Nanorods. Appl. Funct. Mater..

[14] Chien W., Lin C.Y., Tsai S.T., Yang C.F., Chang C.C., Shu S.T. (2018). Using a Thermal Treatment Process to Enhance Anodic Oxidation TiO_2_ Nanotubes with High Degradation Effect of Methylene Blue. Sens. Mater..

[15] Raheb I., Manlla M.S. (2021). Kinetic and thermodynamic studies of the degradation of methylene blue by photo-Fenton reaction. Heliyon.

[16] Al-Zaban M.I., Mahmoud M.A., AlHarbi M.A. (2021). Catalytic degradation of methylene blue using silver nanoparticles synthesized by honey, Saudi. J. Biol. Sci..

[17] Isai K.A., Shrivastava V.S. (2019). Photocatalytic degradation of methylene blue using ZnO and 2%Fe–ZnO semiconductor nanomaterials synthesized by sol–gel method: A comparative study. SN Appl. Sci..

[18] Lv J., Fang M. (2018). Photoluminescence study of interstitial oxygen defects in ZnO nanostructures. Mater. Lett..

[19] Lin B., Fu Z., Jia Y., Liao G. (2001). Defect Photoluminescence of Undoping ZnO Films and Its Dependence on Annealing Conditions. J. Electrochem. Soc..

[20] Purbayanto M.A.K., Nurfani E., Chichvarina O.A., Ding J., Rusydi A., Darma Y. (2018). Oxygen vacancy enhancement promoting strong green emission through surface modification in ZnO thin film. Appl. Surf. Sci..

